# Sarcoidosis with Arteriovenous Malformation in a 15-Year-Old Girl – The Rarest of the Rare

**DOI:** 10.3389/fped.2015.00077

**Published:** 2015-09-22

**Authors:** Iman Qaiser, Kanwal Nayani, Shakeel Ahmed, Rehan Ali, Mehnaz Atiq

**Affiliations:** ^1^Medical College, Aga Khan University, Karachi, Pakistan; ^2^Department of Pediatrics, Aga Khan University, Karachi, Pakistan

**Keywords:** sarcoidosis, AVMs, pulmonary arteriovenous malformations, pulmonary sarcoidosis, complications of sarcoidosis

## Abstract

**Introduction:**

Sarcoidosis is an uncommon multi-system disorder with many possible complications. Arteriovenous malformations (AVMs) are a rare vascular complication of sarcoidosis.

**Case description:**

A 15-year-old girl presented to the Pediatric Clinic at AKUH with pulmonary, hepatic, joint, and skin manifestations. Physical examination and investigations pointed toward sarcoidosis, including raised erythrocyte sedimentation rate, angiotensin converting enzyme (ACE), and alanine transaminase (ALT). An incidental finding of pulmonary arteriovenous malformation (PAVM) was noticed on echocardiography. She responded to oral corticosteroids, her ACE and ALT levels improved. There was lack of indication for pulmonary angio-embolization for her PAVM. On a 3-year follow-up, her condition improved and she is clinically well.

**Discussion:**

Pulmonary arteriovenous malformation is an extremely rare complication of sarcoidosis, especially among the pediatric population. Hence, this is the first reported case of its kind. The relation between sarcoidosis and PAVM is difficult to establish; however, there are some theories. This condition may be treated depending on the symptoms. Since our patient did not have any significant symptoms of PAVM, she was treated for the underlying disease, i.e., sarcoidosis.

**Conclusion:**

While dealing with patients having multi-system disorders like sarcoidosis, one must be very vigilant so as not to miss out on any complication. Regular follow-up visits should be scheduled to rule out new complications and to monitor the past ones.

## Introduction

Sarcoidosis is a rare disorder characterized by non-caseating granulomas in multiple organs. There is no definite known cause (1). Most patients present with lung, eye, and skin manifestations though any part of the body can be involved (2). Varying degrees of severity and complications have been seen among patients. Pulmonary, ocular, and neurological are some of the more common complications found (2). Rarely, vascular complications are seen (2). In turn, arteriovenous malformations (AVMs) are abnormal communications between arteries and veins, most commonly found in the brain (3). Pulmonary AVMs are very rare. It can present with dyspnea, hemoptysis, and platypnea. Most are due to underlying hereditary telangiectasia (4). We report a case of a 15-year-old girl with sarcoidosis found to have pulmonary AVMs, an extremely rare complication.

## Case Presentation

A 15-year-old Pakistani girl reported to our clinic with complaints of generalized rash, painful swollen small joints, and cyanosis since 2 years of age. She also complained of low grade fever, breathlessness, and fatigue. Examination revealed an emaciated, anemic, centrally cyanosed girl with weight and height below 5th percentile. Oxygen saturation was 84% and respiratory rate 24/min. Grade IV clubbing was present. Generalized, erythematous, maculopapular rash was present, mostly on the trunk. Tender, swollen joints were also found with limited range of movement. Abdominal exam showed gross hepatosplenomegaly and positive shifting dullness. Chest and precordium exams were normal. Initial assessment of autoimmune disease, cyanotic heart disease, systemic vasculitis, and failure to thrive was made.

Initial laboratory workup showed hemoglobin (Hb): 11.3 g/dl, hematocrit (HCT): 35.7%, white blood cells (WBC): 6.1 × 10^9^ (PMN: 57.4%, L: 34.5%), Platelets: 306 × 10^9^; all within the normal range. Her erythrocyte sedimentation rate (ESR) was raised (35 mm in first hour), along with alanine transaminase (ALT), which was 164 IU/l, gamma-glutamyl transpeptidase (GGT), which was 148 IU/l, and alkaline phosphatase (AP), which was 553 IU/l. Viral markers for hepatitis (B and C) were non-reactive. Autoimmune profile [anti-nuclear antibody (ANA), anti-DNA, Anti-mitochondrial antibody (AMA), anti-smooth muscle antibody (ASMA), rheumatoid factor (RF), cytoplasmic anti-nuclear cytoplasmic antibody (c-ANCA), and perinuclear anti-nuclear cytoplasmic antibody (p-ANCA)] was negative. Chest x-ray was normal. Ultrasound of abdomen showed an enlarged spleen (13 cm) and an enlarged liver with normal echotexture. Liver biopsy revealed moderate portal lobular inflammation (Grade 3) and peri-portal fibrosis (Stage 2). Skin biopsy revealed histiocytic reaction with multinucleated giant cells with no evidence of telangiectasia or vasculitis, thus suggesting chronic granulomatous disease. Further investigations were done on the lines of sarcoidosis. Expectedly, angiotensin converting enzyme (ACE) level was raised with a value of 100 U/l (normal: <52 U/l); however, serum calcium was normal at 8.9 mg/dl (normal: <10.5 mg/dl). The eye exam did not reveal any abnormality.

**Figure 1:**
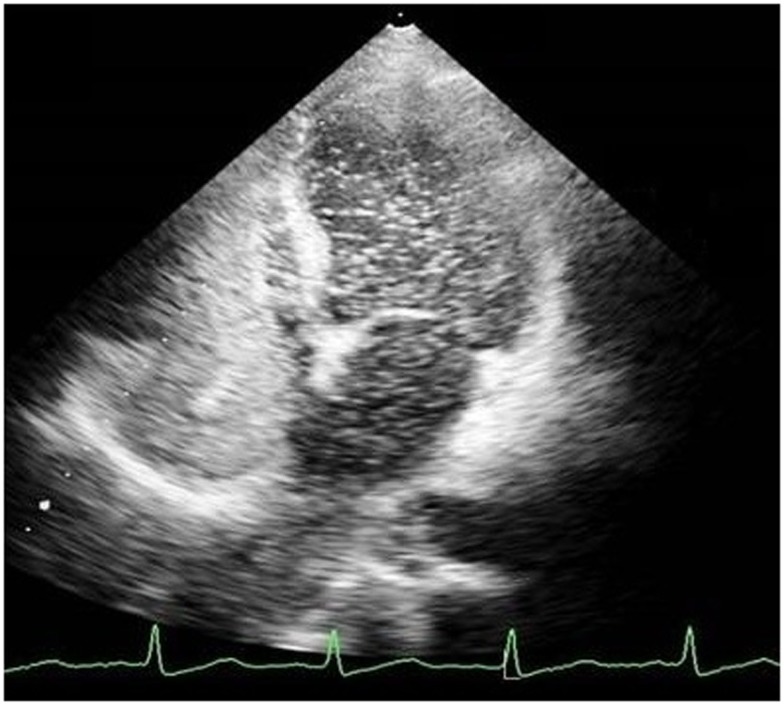
**A pulmonary AVM as seen on echocardiogram**.

**Figure 2:**
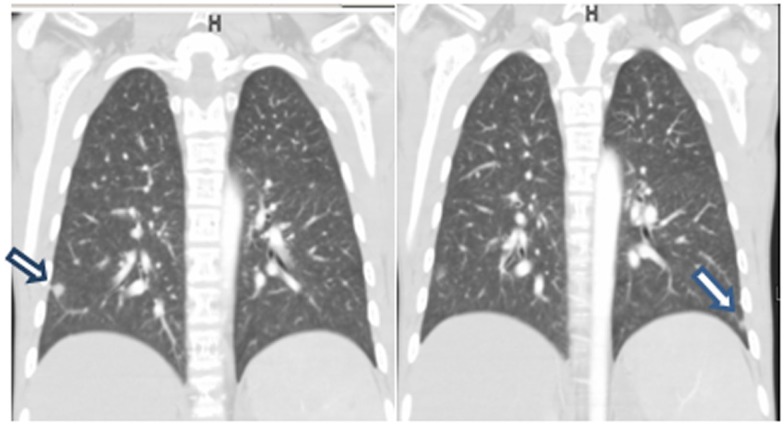
**Coronal CT images showing AVM on right and left side (arrow)**.

Echocardiography was done to investigate cyanosis and breathlessness. Conventional echocardiography ruled out any structural lesions of the heart but bubble echocardiography showed contrast bubbles in left atrium after three cardiac cycles, suggestive of pulmonary AV malformation (Figure 1). CT angiogram was thus done and revealed three soft tissue density nodules. These were in the right upper lobe, right lower lobe, and a smaller one in the left lower lobe. Vessels were seen to be arising from the nodules. No signs of infection of interstitial lung disease were seen (Figure 2). All these findings were complementing pulmonary arteriovenous malformation (PAVM).

Final assessment of sarcoidosis with PAVM was made. She was started on oral steroids and monitored closely. ACE and ALT levels declined significantly over the next few weeks to 53 (normal: <52) and 65 (normal: <35), respectively. Hepatosplenomegaly began to regress. Her cyanosis and exertional dyspnea improved gradually too. It was decided to not carry out pulmonary angio-embolization as she had multiple AV malformations and it was less likely to benefit her. On a 3-year follow-up, she was clinically stable with no active symptoms and her laboratory parameters were back to normal.

## Discussion

Pediatric sarcoidosis is an infrequent condition. A Danish study has reported an incidence of 0.29 cases per 100,000 children as opposed to 5–40 cases per 100,000 adults (5, 6). PAVM is also a unique occurrence and so far no studies have established a pediatric prevalence of the disease, though the prevalence in adults is reported to be 2–3 per 100,000 population (6). Most cases of pediatric PAVM occur in children with underlying hereditary hemorrhagic telangiectasia (5). Thus, to come across a case in which sarcoidosis is complicated by PAVM is extremely rare. In our literature search, we found only one such case reported from Japan of a 27-year-old woman (7). No such pediatric cases have been reported so far and this is the first of its kind.

The relation between sarcoidosis and PAVM is extremely difficult to establish. Both diseases have an unknown etiology (3, 5). Current theories postulate that most PAVMs are caused by defects during vascular development in the fetus, such as aberrant TGF-beta signaling or a defect in terminal arterial loops (3). No theory has thus far suggested a cause for non-congenital PAVMs (3). On the other hand, there are numerous etiological theories for the occurrence of sarcoidosis, including environmental triggers, infectious agents, T-cell abnormalities, and effect of cytokines, such as TNF alpha (8, 9). No common etiological factors for the disease were found, which makes it very difficult to understand how the two disease processes occurred together in our patient. We could theorize, however, that certain pro-inflammatory cytokines released in sarcoidosis could have led to progressive vasodilation of thin-walled capillary sacs, formation of multi-loculated sacs, and finally rupture of intervening vascular walls leading to formation of small PAVMs. Further research is required to establish a relation between the two.

It is also a known fact that cirrhosis has an association with PAVM (5, 10). This association has been termed hepatopulmonary syndrome (HPS) and is defined by liver dysfunction or portal hypertension, intrapulmonary vascular dilations, and abnormal gas exchange (11). The vascular component of HPS may include pulmonary capillary dilatation or macrovascular AVMs (12, 13). In our case, the patient had hepatomegaly and biopsy also revealed signs of moderate cirrhosis, which is another cause of formation of PAVM (14). Thus, we can also postulate that PAVMs developed in this patient secondary to cirrhosis, which itself was a manifestation of the granulomatous processes of sarcoidosis.

Pulmonary arteriovenous malformation can be diagnosed on contrast-enhanced transthoracic echocardiography with saline. In this procedure, saline is injected into a peripheral vein and the subsequent changes in both the atria are noted. In the case of an intracardiac shunt, bubbles will be visualized in the left atrium within one cardiac cycle after their appearance in the right atrium, while with PAVMs bubbles in the left atrium will appear after a delay of three to six cardiac cycles, as was the case in our patient (12). Diagnosis of PAVM is further confirmed by using pulmonary angiography (12).

Clubbing is a sign that indicates severe, chronic, or multiple PAVMs. Review of data based on the six largest consecutive case-series of PAVMs during the past 50 years, revealed that the average incidence of clubbing in PAVMs was 5% (15). Most pathologies causing long-term cyanosis and clubbing are picked up on echocardiography, e.g., intracardiac shunts. However, extracardiac shunts are better picked up on contrast echocardiography. With a sensitivity of 100% and a specificity of 49%, contrast echocardiography is the recommended first investigation of choice to diagnose PAVMs and preferred over any other methods including 100% oxygen method and radionuclide perfusion scanning (15).

Literature has shown that PAVMs do not regress on their own. Most remain stable and 25% enlarge with time (16). The morbidity and mortality are uncertain but it is known that most deaths occur due to stroke, cerebral abscess, or hemothorax (16, 17). That being said, not all PAVMs require treatment. Indications for treatment have been not clearly defined and a final decision is made by comparison of risk of complications versus risk of treatment procedures. In our patient, the CT showed PAVMs that were small in size (6 mm × 5 mm), which did not require any intervention (8, 11, 16). She will be followed with a CT in the future to look for enlargement and to recognize any worsening symptoms. If found then treatment with pulmonary angio-embolization will be the first choice.

## Conclusion

When dealing with a multi-systemic disease, such as sarcoidosis, which can involve almost any part of the body, one must be vigilant while evaluating a patient. While one can be extremely thorough in the evaluation of a patient, there is always a limit and one should keep an open mind of what one may find. The echocardiography done in this case, incidentally found the PAVM. It is important that the physician keep a regular follow-up of patients with PAVM so as to prevent any possible complications.

## Consent

Written informed consent to share information and images in this case report was obtained from the patient prior to publication.

## Conflict of Interest Statement

The authors declare that the research was conducted in the absence of any commercial or financial relationships that could be construed as a potential conflict of interest.

## Funding

This work was supported by the Singapore Immunology Network (SIgN) core grant.
